# Hypothyroidism and brain developmental players

**DOI:** 10.1186/s13044-015-0013-7

**Published:** 2015-02-11

**Authors:** R G Ahmed

**Affiliations:** Division of Anatomy and Embryology, Zoology Department, Faculty of Science, Beni-Suef University, Beni-Suef, Egypt

**Keywords:** Hypothyroidism, Brain development, Transporters, Deiodinases

## Abstract

Most of our knowledge on the mechanisms of thyroid hormone (TH) dependent brain development is based on clinical observations and animal studies of maternal/fetal hypothyroidism. THs play an essential role in brain development and hormone deficiency during critical phases in fetal life may lead to severe and permanent brain damage. Maternal hypothyroidism is considered the most common cause of fetal TH deficiency, but the problem may also arise in the fetus. In the case of congenital hypothyroidism due to defects in fetal thyroid gland development or hormone synthesis, clinical symptoms at birth are often mild as a result of compensatory maternal TH supply. TH transporters (THTs) and deiodinases (Ds) are important regulators of intracellular triiodothyronine (T3) availability and therefore contribute to the control of thyroid receptors (TRs)-dependent CNS development and early embryonic life. Defects in fetal THTs or Ds may have more impact on fetal brain since they can result in intracellular T3 deficiency despite sufficient maternal TH supply. One clear example is the recent discovery of mutations in the TH transporter (monocarboxylate transporter 8; MCT8) that could be linked to a syndrome of severe and non reversible psychomotor retardation. Even mild and transient changes in maternal TH levels can directly affect and alter the gene expression profile, and thus disturb fetal brain development. Animal studies are needed to increase our understanding of the exact role of THTs and Ds in prenatal brain development.

Thyroid hormone (TH) is essential for a number of physiological processes and is particularly critical during nervous system development [1-7]. In developing brain THs stimulate and coordinate processes such as neuronal proliferation, migration, growth of axons and dendrites, synapse formation and myelination [4,7-22]. Disturbance of these processes leads to abnormalities in the neuronal network and may result in mental retardation and other neurological defects, including impaired motor skills and visual processing [23-27]. Hypothyroidism in adults has been associated with mood symptoms and reduced quality of life [16,28,29]. It is estimated that more than 12% of the US population will develop a thyroid condition during their lifetime, and an estimated 20 million Americans have already some form of thyroid disease [30]. Besides, some of the most prominent and common symptoms of thyroid disease are those that result from the effects of TH on the CNS [31].

THs act mainly by binding to nuclear TH receptors (TRs), which are ligand activated transcription factors directly controlling gene expression in neurons and glial cells [11]. They exert their action mainly through binding of 3,5,3′-triiodothyronine (T3) to nuclear receptors that directly influence the expression of TH-regulated genes [16]. Intracellular TH action is therefore dependent on both the availability of T3 and its receptors [8,9,11,12]. TH uptake in cells is regulated by specific TH transporters (THTs) and local activation and inactivation is regulated by deiodinases (Ds) [32]. Generally, intracellular activation or inactivation of L-thyroxine (T4) and T3 in turn is determined by three types of Ds, namely D1, D2, and D3 [24,33-35]. This local production and degradation of T3 is extremely important because it allows regulating intracellular T3 availability in developing brain regions/cell populations at least in part independently of the amount of T3 available in the rest of the body [32]. Before deiodination or binding to TRs can occur, THs have to enter brain cells via specific transmembrane THTs [36], including members of the organic anion transporter family (OATP), L-type amino acid transporters (LATs), Na^+^/Taurocholate cotransporting polypeptide (NTCP), and monocarboxylate transporters (MCTs) (Table 1) [37-39]. Particularly, monocarboxylate transporter 8 (MCT8) has recently been identified as an active and specific THT. Several of these transporters are also localized at the blood-cerebrospinal fluid barrier (BCSFB) and the blood–brain barrier (BBB) and regulate TH uptake from the general circulation at the level of the choroid plexus and the capillaries throughout the brain [40-43].

**Table 1:** **Types of thyroid hormones transporters and their iodothyronine derivates** [16]

**Transporter** ^***a***^	**Iodothyronine derivates**	**Specificity** ^***b***^
MCT8	T3, T4, rT3, T2	+++
MCT10	T3, T4	++
OATP1A1	T3, T4, rT3, T2, T4S, T3S, rT3S, T2S	+
OATP1A2	T4, T3, rT3	
OATP1A3	T4, T3	
OATP1A4		
OATP1A5		
OATP1B1	T4, T3, T3S, T4S, rT3S	
OATP1B2	T3, T4	
OATP1B3	rT3, T4S, T3S, rT3S	
OATP1C1	T4, rT3, T3, T4S	++
OATP2B1	T4	+
OATP3A1 (V1/V2)		++
OATP4A1	T3, T4, rT3	+
OATP4C1	T3, T4	
OATP6B1		
OATP6C1		
LAT1	T3, T4, rT3, T2	
LAT2		
NTCP	T4, T3, T4S, T3S	++

^*a*^The human protein symbol is presented, if TH transport has been demonstrated in different species including humans. ^*b*^If a transporter only transports iodothyronine derivatives, specificity is high (+++). If fewer than five other ligands are known, specificity is moderate (++). If more than five ligands are known, the transporter is denoted as multispecific (+).

The importance of THTs for brain development was clearly demonstrated when researchers were able to link mutations in the human *SLC16A2* gene coding for MCT8 to a previously described form of X-linked psychomotor retardation, the Allan-Herndon-Dudley syndrome [44,45]. All patients have a severe mental retardation, they cannot speak and most of them are unable to sit upright, crawl, stand or walk. Their thyroid phenotype is quite abnormal with a substantial decrease in plasma T4 and a more than twofold increase in plasma T3 as well as an increase in thyroid-stimulating hormone (TSH). Notably, preterm infants show transient hypothyroxinemia without TSH elevation [46]. In addition, the degree of neurodevelopmental delay in preterm infants becomes severe according to the decreasing gestational age. Epidemiological and animal studies had shown that maternal subclinical hypothyroidism had significant negative impact on neurodevelopment [19,22,47]. To gain further insight in the pathogenic mechanisms underlying these diseases, MCT8 knockout (KO) mice have been generated [42,48]. The serum TH parameters in MCT8 KO mice are affected in the same way as in human patients but surprisingly they seem to develop normally, without overt neurological phenotype. Further analysis showed that the entry of T4 into the brain was not disturbed while uptake of T3 was diminished; suggesting that MCT8 is very important for T3 transport across the BBB and/or BCSFB while the passage of T4 is facilitated by other transporters [49]. The overall T4 and T3 content of cerebrum and cerebellum were decreased by more than 50%, but detailed morphological studies as well as analysis of T3 target genes showed that neuronal uptake of T3 was impaired in an area- or even cell-specific manner. There were signs of a pronounced hypothyroid status in the thyroid-releasing hormone (TRH) producing neurons of the hypothalamic paraventricular nucleus, a mild hypothyroid status in RC3 expressing neurons of the striatum but an apparently normal thyroid status in several cell types in the cerebellum [42]. It was concluded that in mice, more than in humans, other THTs can compensate for the lack of MCT8 in neurons, thereby protecting the brain from severe neurological deficits. The critical restriction to T3 transport in the absence of MCT8 could be located at the BBB rather than the plasma membrane of individual neurons [50]. This agrees with the hypothesis that the high OATP1C1 expression in cerebral microvessels in rodents, as opposed to the situation in humans, contributes to the mild phenotype in MCT8 null mice [51]. Recently also a combined MCT8- and D2-deficient mouse has been described. Analysis of 34 target genes in the cerebral cortex of single and double KO mice demonstrated that the expression of only 3 of them was affected by the elimination of MCT8 alone while this number increased to 24 when both MCT8 and D2 were absent, suggesting that D2 can partly compensate for the decrease in TH uptake in MCT8 deficient mice [52]. While several groups have studied the exact localization of THTs at the level of the BCSFB in the choroid plexus and at the level of the BBB in both macro- and microvessels [51,53], only a few detailed reports are available on their cell-specific distribution in other regions of the brain. In situ hybridization (ISH) for MCT8 in murine brain showed the highest signal in several layers of the cerebral cortex and the hippocampus, in the amygdala, and many basal ganglia. In the cerebellum, expression was predominantly in the Purkinje cell layer. Using specific markers it could be shown that MCT8 is predominantly expressed in neurons, and to some extent in the tanycytes lining the third ventricle [50,54,55]. Analysis of primary murine cell cultures demonstrated high levels of MCT8 protein in neurons and low levels in astrocytes and oligodendrocytes. Importantly, MCT8 deficiency has important metabolic consequences in the brain that could not be correlated with deficiency or excess of TH supply to the brain during adulthood [56]. Also, Müller et al. [57] indicated that MCT10 indeed participates in tissue-specific TH transport and also contributes to the generation of the unusual serum TH profile characteristic for MCT8 deficiency. LAT1 showed the same expression pattern as MCT8 [35]. MCT8 deficiency has important metabolic consequences in the brain that could not be correlated with deficiency or excess of TH supply to the brain during adulthood [56]. In human late fetal brain LAT2 was found only in microglia while in perinatal mice it was also widely expressed in neurons and astrocytes [35,55]. Information on the ontogenetic changes in THT expression in mammalian brain is quite scarce, especially for early developmental stages. In birds, THTs have been studied first in quail in relation to their possible involvement in the regulation of photoperiodicity at the level of the hypothalamus. Four members of the OATP family were found in choroid plexus: OATP1A1, OATP1B1, OATP1C1, and OATP3A2. The ventro-lateral walls of the basal tuberal hypothalamus showed strong expression of OATP1C1 and weak expression of OATP1B1 [58]. Analysis of chicken OATP1C1 substrate specificity in a cell culture system showed that the protein is a highly specific transporter for T4 (Km 6.8 nM) [58]. While MCT8 mRNA levels increased gradually between embryonic day (E) 4 and E10, OATP1C1 expression showed a divergent pattern. The overviews presented here are consistent with the evolving view that the importance of THTs for proper brain development [59].

On the other hand, deiodination is an important step in the activation and inactivation of THs (Table 2) [11,16]. D3 is essential for normal zebrafish embryonic and early larval development [60]. D2 and D3 are the main Ds expressed in brain and their distribution pattern has been analyzed in a few vertebrate species. The general picture appearing from studies in rodents is that D2 is expressed primarily in glial cells while D3 is expressed in neurons [24,61]. In rat forebrain D2 is highly expressed in the external layers of the cerebral cortex and in the hippocampus while in cerebellum the signal is predominantly found in the granular layer. Co-expression studies with Glial fibillary acidic protein (GFAP) confirmed that in both brain regions D2 is present in astrocytes and that it is also highly expressed in ependymal tanycytes lining the third ventricle [61]. In forebrain of the newborn rat D3 seems to be restricted to a few specific regions such as the bed nucleus of the stria terminalis and the amygdala but this expression is no longer observed at postnatal day 10 [62]. In human fetal cerebral cortex samples both D2 and D3 were detected as early as 7–8 weeks of gestation [63]. Up to 20 weeks, D2 activity was rather high and comparable in cerebral cortex and cerebellum. In contrast, D3 activity was much higher in cerebellum than in cerebral cortex and levels in cerebellum declined towards mid-gestation [64]. Expression of Ds has also been studied in avian brain, mainly in chicken and quail [65]. A few research groups investigated Ds expression in chicken brain during embryonic development. Messenger RNA for all three Ds is present in E4 brain [41] but for D1 this is not accompanied by detectable activity throughout embryonic development [66]. Both D2 mRNA and activity increase gradually in all brain regions up to the day before hatching and then decrease again [41,66,67]. For D3 mRNA the pattern varies more between different brain regions and activity levels do not always parallel expression data [41,66]. At the cellular level clear D2 protein staining was detected in the ependymal cells of the choroid plexus, the tanycytes of the third ventricle and the Bergmann glial cells in cerebellum at E18 [68-70]. The D3 protein was abundantly present in ependymal cells of the choroid plexus in the days around hatching, and in cerebellar Purkinje cells 1 day posthatch (C1), but not yet at E18 where a weak signal was observed in the external granular layer [71]. Polymorphisms in D2 have been associated with changes in circulating TH levels [72] and with bipolar disorder [73]. No mutations in Ds leading to an overt neurological phenotype, as for MCT8, have been discovered so far. KO mice have been generated for all three Ds, alone or in combination [74-78]. In chicken, OATP1C1, MCT8 and D3 are expressed in the choroid plexus and its precursors allowing selective uptake of THs at the blood-cerebrospinal fluid-barrier with subsequent inactivation of excess hormone [79]. In contrast, the developing BBB does not express OATP1C1 or MCT8 but appears to be a site for TH activation by D2. Expression of D3 in several sensory brain centers may serve as protection against premature TH action. Expression of D2 and MCT8 but not D3 in the developing pituitary gland allows accumulation of active THs even at early stages. MCT8 is widely expressed in grey matter throughout the brain [79]. At first sight, none of these mice presented a clear neurological phenotype and only the D3 KO mice showed problems in reproduction and fetal survival. D1 KO and D2 KO mice have normal circulating levels of T3 and no compensatory changes were found in D2 or D3 activities in the brain. Although the T3 content in brain of D2 KO mice is substantially reduced, the expression of several TH responsive genes is not or only mildly affected when compared with hypothyroid mice [80]. D2 KO mice do, however, show hearing loss and a retarded and abnormal development of the cochlea [81]. The effects observed in D2 KO mice were intensified following combined knockout of D1 and D2. More detailed analysis of their neurological functions showed no differences in reflexes but some reduction in agility and possibly some problems with vision [32]. While D3 KO mice have low circulating levels of T4 and T3 during most of their life, perinatal mice have markedly elevated serum T3 levels. Their brain shows signs of thyrotoxicosis with high T3 levels and increased expression of TH responsive genes in the first days after birth. This changes to a hypothyroid status by the time of weaning but in old D3 KO mice the brain again turns to a thyrotoxic state despite reduced serum TH levels. It has been shown that the transition from a thyrotoxic to a hypothyroid status does not occur at the same time in all brain regions [78,82]. D3 KO mice also have impaired hearing, showing that both premature and delayed cochlear maturation due to respectively T3 surplus or deficiencies have permanent adverse impact on hearing [83]. Further analysis of D3 KO mice also demonstrated that D3 controls survival and maturation of cone photoreceptors and that D3 KO mice loose the majority of cones in the developing retina through neonatal cell death [84]. TH signaling exerts regulatory roles in early *Xenopus laevis* neurogenesis and second, that this period represents a potential window for endocrine disruption [85]. In rodents and humans, almost all T3 found in the fetal cerebral cortex is generated through local deiodination of circulating maternal T4 [64,86,87]. The fetal dependence on maternal T4 is due **(i)** to the late development of the fetal thyroid gland (in rodents thyroid function begins by E17–18 and in humans by the 18–20 gestational week) and **(ii)** to the increased activity of D2 and D3 in placenta and fetal tissues [34,87-89]. As a consequence of the increased activity of Ds in the fetus, serum T3 levels are maintained low and the local generation of cerebral T3 from T4 is enhanced [33,64,87]. To respond to this requirement, there is an estrogen-dependent increase of maternal thyroid function that transiently induces an increase of **(i)** circulating thyroxine-binding globulin, affecting the T4 extra-thyroidal pool, and of **(ii)** human chorionic gonadotropin, transiently stimulating thyrocytes [90]. This increased maternal thyroid function consequently needs increased iodine intake. In the general population, even small variations in maternal thyroid function during pregnancy may affect the developing head of the young child [21].

**Table 2:** **General characteristics of the iodothyronine deiodinases** [16]

**Characteristic**	**D1**	**D2**	**D3**
Reaction kinetics	Ping-pong	Sequential	Sequential
Reaction catalyzed (Deiodination)	5 or 5' (ORD+IRD)	5' (ORD)	5 (IRD)
Main form	T4-T3, rT3- T2	- T4- rT3, T3- T2	- T4- rT3- T2
Substrate preference	5: T4S>T3S>>T3, T4	T4>rT3	T3>T4
	5': rT3, rT3S>T2S>>T4		
Sulfation of substrates	Stimulation	Inhibition	Inhibition
Substrate limiting *K*M	0.5 mM	1–2 nM	5–20 mM
In vitro cofactor limiting *K*M	1–10 Mm DTT	>10 mM DTT	=70 mM DTT
Molecular mass (kDa)	29	30	32
Selenocysteine	present	present	present
Homodimer	Yes	Yes	Yes
Location	- Liver, kidney, thyroid and pituitary.	- Pituitary, brain, BAT, thyroid^a^, heart^a^ and skeletal muscle^a^.	- Brain, skin, uterus, placenta, fetus and in other sites of the maternal- fetal interface, such as the umbilical arteries and veins.
Subcellular location	- Liver: endoplasmic reticulum. - kidney: basolateral plasma membrane	- Microsomal membranes	- Microsomal membranes
Functions	- Production serum T3 and the clearance of serum rT3.	- Catalyzes the outer ring deiodination of T4 to T3 and is thus important for the local production of T3.	- Catalyzes the inner ring deiodination of T4 to rT3 and of T3 to 3,3'-T2.
Activity in hypothyroidism	- Decrease in liver and kidney.	- Increase in all tissues.	- Decrease in brain.
	- Increase in thyroid.		
Low-T3 syndrome	- Decrease	- No change	- No change
Active site residues	- Selenocysteine histidine and phenylalanine.	- (Seleno-)cysteine?	- Selenocysteine
Human gene structure and location	- 1p32-p33, 17.5 kb and 4 exons.	- 14q24.3, 2 exons and 7.4-kb intron.	- 14q32
Promoter elements	- TRE, RXR, no CAAT or TATA box.	--	--
Propylthiouracil inhibitor	++++	+	+/-
Aurothioglucose inhibitor		++	++
Iopanoic acid inhibitor	+++	++++	+++
Thiouracils	++++	-/+	-
Iodoacetate		+	?
Flavonoids	+	+++	+++

^a^Humans only. T2 is diiodothyronine, T3 is triiodothyronine, rT3 is reverse triiodothyronine, T4 is thyroxine, T2S is diiodothyronine sulfate, T3S is triiodothyronine sulfate, T4S is thyroxine sulfate, rT3S is reverse triiodothyronine sulfate, ORD is outer ring deiodination, IRD is inner ring deiodination, TRE is T3-responsive element, RXR is retinoid X receptors and DDT is dithiols.

From the information above it is clear that THTs and Ds are expressed in brain in a region- and cell specific way (Table 3). It is also clear that deficiencies in THTs or Ds can adversely affect neurodevelopment but the neurological phenotypes are far from understood. The defects are partly different from the ones observed due to fetal hypothyroidism and the phenotype can also differ between humans and rodents. It seems therefore essential to extend the research in two directions, towards earlier developmental stages and towards other model species. Additional research in different models using conditional silencing will hopefully further improve our understanding on how THTs, Ds and TRs cooperate to regulate TR-mediated impact on vertebrate CNS development.

**Table 3:** **General summary about the developmental thyroid hormone mechanisms (deiodinases, transporters, sulfotransferases and receptors) in human, rat and chicken** [16]

**Human**		**Rodent (rat)**		**Chicken**	
**Week post conception**		**Day post conception**		**Incubation day**	
1 W	- D3 is detected in uterine wall.	1 GD	D2 and D3 are observed in uterine wall.	5 h (blastula stage)	- TRα mRNA is noticed and the levels markedly increased during neurulation.
3 W	- Thyroid gland begins.	7 -8.5 GD	- Time of implantation process.	24 h	- mRNA levels of D1, D2, and D3 are detected in whole embryos.
			- Very high D3 activity is detected in decidual tissue.		
4-6 W	- TBG is observed in thyroid follicle cells at GD 29.	9 GD	- Thyroid gland is first visible as an endodermal thickening in the primitive buccal cavity.	48 h	- OATP1C1 expression appears.
	- TRH is detected in fetal whole-brain at 4.5 weeks of gestation.		- TH is detected in rat embryotrophoblasts		
	- T4 is transferred *via* the placenta and has been found in the gestational fluid sac from 4 to 6 W.				
5-11 W	- Maternal-embryo transfer of THs has been detected in embryonic coelomic fluid and amniotic fluid.				
	- All the mRNAs encoding THTs are expressed in placenta from 6 W and throughout pregnancy.				
8 W	- T4, T3 and rT3 are detected in coelomic/amniotic fluids.	10 GD	- T4, T3 and TRβ are detected in embryo/trophoblast unit.	E2-E4	- T3, THTs, Ds and TRs are expressed in whole embryos.
	- TRs, D2 and D3 are noticed in fetal brain.				
10 W	- TSH is first detected in the fetal pituitary.			E4	- OATP1C1 expression is more than 10-fold higher in the telencephalon and diencephalon compared to the mesencephalon and rhombencephalon.
					- D2 mRNA levels are highest in the diencephalon.
8-10 W	- The fetus is able to produce THs during this period, but prior to that time, is totally dependent on maternal THs.			E5	- TRα mRNA is widely distributed in fore-, mid- and hind-brain.
11 W	- TBG levels are detected in fetal serum and increased through gestation.			E6	- T4 and T3 are detected in embryonic brain.
8-11 W	- TRH is detected in fetal hypothalamus.			E7	- D2 activity is observed in the brain before the onset of thyroid function and increases significantly.
12 W	- T4 and T3 are observed in serum and brain.	13 GD	- Placental circulation established.	E8	- D2 mRNA is noticed in cell clusters throughout the brain, particularly in rhombencephalon.
	- Total serum T4 and T3 are low, free T4 is relatively high.		- TRs and TH are observed in fetal brain.		- OATP1C1 levels are declined substantially in all brain regions.
	- rT3 is noticed in serum relatively high.		- D3 and D2 are detected in uterus and placenta.		
	- TH synthesis begins in fetal thyroid.				
	- Decreased mRNA expression of OATP1A2 but no change for OATP4A1 at 9–12 W compared to term.				
14 W	- Expressions of mRNAs encoding MCT8, MCT10, OATP1A2 and	14 GD	- TRH mRNA is detected in neurons of the fetal hypothalamus.	E4-E8	- D3 mRNA levels are markedly different in the telencephalon and diencephalon but remain stable, while the levels in mesencephalon and rhombencephalon show a sharp decrease and increase, respectively, during these days.
	LAT1 are significantly lower prior to 14 W compared to term				
		15 GD	- Pituitary TSH mRNA expression begins.	E9-10	- Several elements of the TH action cascade are observed in the brain of embryos long before their own thyroid gland starts hormone secretion.
			- TRH mRNA is detected in the developing paraventricular nuclei of the hypothalamus.		
16 W	- D3 is observed in placenta and fetal epithelial cells.	16-19.5 GD	- TRs are observed in liver, heart and lung.	E10	- The thyroid gland is fully functional.
	- D3 and TRs are detected in fetal liver.		- D1 and D2 are noticed in fetal tissues.		
	- D1 is noticed in heart and lung.		- TRH is produced in low levels in hypothalamus and increases approximately threefold by GDI9.5.		
	- Significant fetal TH secretion begins.				
16-20 W	- Duplication of TBG concentrations.	17 GD	- TH synthesis begins in fetal thyroid	E13	- Brain D2 is elevated at the peak of neuroblast proliferation.
			- TSH protein and Sulfotransferase are observed.		
		18 -22 GD	- The total T4 and T3 concentrations in fetuses are increased dramatically because of maturation of hormone synthesis of the fetal thyroid gland.	E14	- The strong increase in intracellular T3 has been observed.
			- The coordination between THTs and Ds is regulated both transplacental TH passage from mother to fetus and the development of the placenta itself through the progress of gestation.		
20 W	- A steady increase in serum TH levels begins and continues to term.	19 GD	- Significant fetal TH secretion begins.	E15	- Plasma T4 levels start rising markedly around this day.
			- Marked rise in serum TH but levels at birth still below those in adult.		
22-32 W	- Serum total and free T4 and T3 near and below adult levels, respectively.	22 GD	- Birth state.	E16	- The decrease in DI activity in gonads is combined with the relatively high D3 activity.
			- Thyroid system is less developed.		
	- The HPT axis begins to mature during the second half of gestation.		- As much as 17.5% of THs found in the newborn are of maternal origin.		- A significant increase in T3 production and in D2-activity and -mRNA expression are combined with a decreased in D3 activity.
	- LAT1 and OATP4A1 have been localized only during the third trimester.				
40 W	- Birth state.	10 PND	- Brain development equivalent to human birth.	E13/14–E17 (synaptogenesis)	- Brain D2 activity is moderately elevated, whereas D3 activity and mRNA expression are highest between these days, followed by a dramatic decrease thereafter.
	- Complete maturation of thyroid system.				
	- MCT8 has been localized in the placenta in all three trimesters of pregnancy.				
	- High concentrations of the different iodothyronine sulfates, T4S, T3S, rT3S and T2S, have been documented in human fetal and neonatal plasma as well as in amniotic fluid during the pregnancy.	10-20 PND	- Serum TH levels continue to rise and are higher than adult levels between these days.	E18	- D1 and D3 are expressed in the granule cells, whereas D2 is found mostly in the molecular layer and the Purkinje cells at that time.
		14-50 PND	- The levels of pituitary and serum TSH slowly decrease from PND 14–16 until reaching adult levels at PND 40.	E19	- The increase in brain T3 production correlates with the appearance of TRβ expression in the cerebellum, telencephalon and optic lobes.
			- TRH levels increase to adult levels by PNDI7–29, then decrease transiently between PND 31–41; adult levels are once again reached at PND 50.		
				E20 (at the moment of pipping)	- The brain is quite well developed at the time of hatching.
			- Adult TRH mRNA expression patterns are observed at PND 22.		- The gradual increases in plasma T4 and hepatic D1 are detected.
					- D3 levels are decreased in spleen and increased in skin and the lungs towards hatching.
					- T3 production seems to be elevated markedly in liver.
					- The rise of T4 is much more pronounced than in plasma.
					- Diminished T4 sulfation is detected.
		30 PND	Complete maturation of thyroid gland.	E14-E19/20	- The T3 breakdown capacity by D3 is high in liver but low in kidney.
				E15/16-E20	- T4 levels in plasma increase gradually during these days.
					- In contrast to TRα expression which increases gradually towards hatching, expression of TRβ shows an abrupt elevation in late development, especially in the cerebellum.
					- The majority of tissues express D3 together with either D1 or D2.
				E17-E20	- The levels of D3 activity noticed in liver are rapidly drop by more than 90%.
					- D1 levels in testis and ovary strongly decrease around hatching.
				E18–E20	- Brain D2 activity is moderately decreased, whereas D3 activity is low.
				E19-E20	- The low T3/T4 ratio is associated with high T3 breakdown in liver and with high T4 inactivation or T3 secretion in kidney.
				E20-C0	- D1 activity gradually increases, reaching a maximum around these period, and decreases slowly to posthatch levels thereafter.
				C1 (first day posthatch)	- The expression of D1 is limited to the mature granule cells and that of D3 to the Purkinje cells exclusively, whereas D2 remains clearly noticed in the molecular layer.
				C2	- Highest D1-activities and -mRNA expressions are detected in the liver, kidney, and intestine.
				C1-C7	- The circulating T3/T4 ratio started to increase gradually during the first week after hatching.

- *Abbreviations:* *W* is week, *GD* is gestation day, *E* is incubation day, *PND* is postnatal day, *C* is posthatch day, *THs* is thyroid hormones, *TRH* is thyroid releasing hormone, *TSH* is thyroid stimulating hormone, *THTs* is thyroid hormone transporters, *MCT* is monocarboxylate transporter, *OATP* is organic anion transporter, *Ds* is deiodinases (D1, 2, 3), *TRs* is thyroid hormone receptors (TRα, β), *T4* is Thyroxine, *T3* is triiodothyronine, *rT3* is reverse triiodothyronine, *T2S* is diiodothyronine sulfate, *T3S* is triiodothyronine sulfate, *T4S* is thyroxine sulfate, *rT3S* is reverse triiodothyronine sulfate, *HPT* is hypothalamic-pituitary-thyroid axis and *TBG* is thyroxin binding globulin.
